# Recent advancements in machine learning for bone marrow cell morphology analysis

**DOI:** 10.3389/fmed.2024.1402768

**Published:** 2024-06-14

**Authors:** Yifei Lin, Qingquan Chen, Tebin Chen

**Affiliations:** ^1^The Second Affiliated Hospital of Fujian Medical University, Quanzhou, Fujian, China; ^2^The School of Basic Medical Sciences, Fujian Medical University, Fuzhou, Fujian, China; ^3^The School of Public Health, Fujian Medical University, Fuzhou, Fujian, China

**Keywords:** machine learning, bone marrow cell morphology, artificial intelligence, deep learning, visualization, automatic classification, automatic identification

## Abstract

As machine learning progresses, techniques such as neural networks, decision trees, and support vector machines are being increasingly applied in the medical domain, especially for tasks involving large datasets, such as cell detection, recognition, classification, and visualization. Within the domain of bone marrow cell morphology analysis, deep learning offers substantial benefits due to its robustness, ability for automatic feature learning, and strong image characterization capabilities. Deep neural networks are a machine learning paradigm specifically tailored for image processing applications. Artificial intelligence serves as a potent tool in supporting the diagnostic process of clinical bone marrow cell morphology. Despite the potential of artificial intelligence to augment clinical diagnostics in this domain, manual analysis of bone marrow cell morphology remains the gold standard and an indispensable tool for identifying, diagnosing, and assessing the efficacy of hematologic disorders. However, the traditional manual approach is not without limitations and shortcomings, necessitating, the exploration of automated solutions for examining and analyzing bone marrow cytomorphology. This review provides a multidimensional account of six bone marrow cell morphology processes: automated bone marrow cell morphology detection, automated bone marrow cell morphology segmentation, automated bone marrow cell morphology identification, automated bone marrow cell morphology classification, automated bone marrow cell morphology enumeration, and automated bone marrow cell morphology diagnosis. Highlighting the attractiveness and potential of machine learning systems based on bone marrow cell morphology, the review synthesizes current research and recent advances in the application of machine learning in this field. The objective of this review is to offer recommendations to hematologists for selecting the most suitable machine learning algorithms to automate bone marrow cell morphology examinations, enabling swift and precise analysis of bone marrow cytopathic trends for early disease identification and diagnosis. Furthermore, the review endeavors to delineate potential future research avenues for machine learning-based applications in bone marrow cell morphology analysis.

## Introduction

It has been reported that cytomorphometric analysis requires only images and not blood samples, making it suitable for low-cost and remote diagnostic systems (1). Yet, the prevailing blood testing devices utilized for disease diagnosis predominantly rely on traditional manual image processing methods to detect bone marrow cells. These methods entail a series of labor-intensive tasks including preprocessing, segmentation, feature extraction, feature selection, and classification (2). Notably, these processes are time-consuming, financially burdensome, lack reproducibility, and exhibit considerable variability among and within hematologists due to differences in expertise, experience, and the subjective nature of manual review (3). Moreover, traditional methods necessitate the complete appearance of the object for accurate recognition. Laser-based cytometry equipment, often employed in laboratories, is costly and demands meticulous hardware calibration and actual blood samples. Hence, there persists a pressing need for cost-effective and robust automated systems capable of bone marrow cell morphology testing and diagnosis (4).

Deep learning, particularly convolutional neural networks (CNNs), has emerged as a prominent tool in applied artificial intelligence (AI), finding widespread application in medical imaging computer-aided systems. Its utilization in the detection, classification, and diagnosis of myeloid cells has shown significant progress. In contrast to traditional image classification methods, deep learning obviates the need for manual feature extraction from bone marrow cell images. Instead, it autonomously extracts abstract, high-level semantic features from input images, thereby enhancing the efficiency of feature learning and extraction (5). Consequently, deep learning has garnered remarkable achievements and witnessed substantial advancements in the medical domain (6). The advent of deep learning has catalyzed significant advancements in computer vision applied to bone marrow cell morphology, particularly in detection and recognition tasks, resulting in marked improvements in accuracy that surpass traditional manual methods. Concurrently, AI and big data processing, employing machine learning-based automated algorithms, have been progressively integrated into the domain of bone marrow cell morphology detection and comprehensive data analysis. Recent studies have underscored their superiority over conventional image processing and analysis techniques (2, 7, 8). Furthermore, leveraging disease characteristics and hematologists’ expertise, AI and big data processing techniques can extrapolate, extend, and simulate data to correlate with disease differential diagnosis, treatment efficacy evaluation, and prognosis assessment. This approach yields efficient and precise intelligent analyses, enabling comprehensive interpretation of big data test results. Such advancements hold profound implications for the future of bone marrow cytomorphometry testing (9). Multiple studies have also demonstrated that machine learning methods combined with digital pathology methods can be used as a robust and rapid screening diagnostic tool to derive new diagnostic features/algorithms from digital bone marrow cell images to optimize classification results (3). As an interdisciplinary technology integrating digital image processing, blood smear image analysis, computer science, and AI, image processing-based computer-aided detection and diagnostic systems have gained widespread usage. They assist hematologists in achieving more precise and standardized analyses of blood cell images (5). This review aims to provide a multidimensional overview of the current automated systems employed for the detection, segmentation, identification, classification, enumeration, and diagnostic evaluation of bone marrow cell morphology. Additionally, it will discuss recent research, pertinent considerations, and future directions in the application of machine learning within this domain.

## Automation of bone marrow cell morphology detection

The conventional approach to myeloid cell detection historically relied on recognizing the complete object’s appearance, a method prone to time inefficiency and bias (2). Detecting bone marrow cells presents additional challenges, given their susceptibility to physical contact, non-uniform background noise, and considerable variation in size and shape (10). Furthermore, specialized training of technicians within hospitals to conduct these tests contributes to healthcare costs and time expenditure. Hence, there is a pressing need for the automation of bone marrow cell morphology testing (6). Moreover, achieving efficient and robust bone marrow cytomorphometry is crucial for numerous biomedical image analysis methods and computer-aided diagnoses (10). In recent years, machine learning frameworks have exhibited promising efficacy and efficiency in processing digital morphological images for various applications.

A study conducted by Chandradevan et al. (11) highlights the potential of machine learning algorithms in detecting bone marrow cells, utilizing a training dataset comprising cellular components from bone marrow aspirates. This emerging technique shows promising results in its early stages. Wang et al. (12) developed an efficient hierarchical deep learning model capable of swiftly localizing bone marrow particles and cell trails, generating regions of interest for subsequent analysis. The framework exhibited superior performance in recall, accuracy, computation time, and generalizability.

Another study by Song et al. (13) proposed a simultaneous deep autoencoder network within a single architecture, enabling accurate detection and classification of irregularly shaped cells in bone marrow histology images. It uses a curve-supported Gaussian model to compute a probability map, which allows for the accurate detection of irregularly shaped cells. In addition, the network contains a novel neighborhood selection mechanism to improve the classification accuracy. This network surpasses traditional methods involving two independent deep learning networks, proving to be more competitive. Several studies based on CNNs (2, 14) suggest that computer-aided automated systems, particularly deep CNNs, can accurately localize leukocyte types in blood images. Additionally, pre-trained neural networks like AlexNet demonstrate exceptional sensitivity, specificity, and accuracy (≥98%) in detecting acute lymphoblastic leukemia subtypes.

Xie et al. (10) introduced a novel structured regression model based on a full residual CNN, capable of generating dense neighborhood maps through learning. These maps exhibit heightened response near the cell center, facilitating efficient and accurate cell detection. Furthermore, Piuri and Scotti (15) demonstrated that extracting morphological indices from microscopic color images enabled automated leukocyte detection. Color spatial light interferometry microscopy (cSLIM), combined with deep learning techniques, serves as a highly sensitive quantitative phase imaging (QPI) method for localizing leukocytes in blood smears. Integrating QPI label-free data with AI, such as phase imaging with computational specificity (PICS), enables precise cell-specific extraction and rapid leukocyte detection (16). Lastly, leveraging deep learning for visual target identification offers novel approaches to myeloid cell detection (17).

## Automated bone marrow cell morphology segmentation

Cell segmentation serves as a crucial preliminary stage for subsequent cell feature extraction and classification (18). However, due to the notably higher cell density in bone marrow smears compared to peripheral blood smears, cells in bone marrow samples tend to be more densely packed and viscous, posing challenges for accurate separation, thus impeding cell interpretation (19). In the realm of automated bone marrow cell identification, the efficacy of cell segmentation significantly influences subsequent processes, including cell classification, identification, and disease diagnosis (10). Computerized imaging systems typically commence with a segmentation phase, and the efficacy of the system hinges significantly on the precision of this initial segmentation process. Consequently, researchers have devoted considerable efforts to refining segmentation algorithms (20). Importantly, segmentation algorithms remain unaffected by factors such as variations in background, staining techniques, and morphological disparities among bone marrow cells, thereby effectively addressing the myriad challenges associated with bone marrow cell segmentation and meeting the demands of clinical applications (10).

Efficient and precise segmentation of color images has been a central focus in computer vision and image analysis, owing to its inherent complexity. Leveraging spatial information derived from pixel color data and pixel adjacency, a segmentation method based on the HSI color space was proposed at an IEEE conference. This color image segmentation algorithm demonstrates superior efficiency, accuracy, and image recognition quality compared to similar approaches (21). The marker-controlled watershed algorithm, a robust image processing technique, finds widespread application in segmenting dense cell images. The algorithm improves segmentation performance, resulting in high accuracies for both sparsely distributed normal white blood cells and dense leukemic cell clusters (7, 20). Arslan et al.’s (20) study introduced a novel segmentation algorithm based on color and shape, designed specifically for segmenting leukocytes in bone marrow images. This algorithm demonstrates superior segmentation performance and high accuracy, accommodating both sparsely and densely distributed cell clusters. By integrating color and shape information at various stages, the algorithm exhibits enhanced robustness in segmenting isolated normal leukocytes as well as merged leukemia cell clusters. Consequently, future research could explore extracting texture features from segmented cells and refining subsequent processing stages by leveraging texture information to mitigate false positive cell identifications.

Many cell segmentation algorithms rely on mathematical morphology and operations within color spaces. In a recent study, a bone marrow cell segmentation algorithm integrating sparse representation and mathematical morphology operations was proposed. This approach harnesses prior knowledge of images alongside sparse representation techniques to achieve precise detection and segmentation of bone marrow cells. Moreover, given the distinctive features of adherent cells in bone marrow smears, a multi-directional mathematical morphological operation was employed to effectively separate these cells (10).

Su et al. (18) introduced a segmentation approach for distinguishing nuclei and non-nuclei within plaques through the application of weighted thresholds derived from stepwise averaging and Otsu’s method. Additionally, they utilized color attenuation transformations, enhanced region growing techniques, and the K-Means algorithm to segment cytoplasm (7). Notably, the segmentation efficacy of cells achieved through K-Means clustering showed improvement irrespective of whether images were cropped or not, thus establishing a groundwork for subsequent cell feature extraction and recognition processes.

Song et al.’s (22) study introduced a novel framework designed to efficiently outline the nucleus and cytoplasm of bone marrow cells within digitized images of bone marrow aspiration biopsies. Initially, the framework delineates megakaryocyte nuclei through a supervised machine learning approach, leveraging color and texture features. Subsequently, for delineating the boundaries of megakaryocyte cytoplasm, a two-channel active contour model employing different deconvolutional staining channels is employed. Compared to alternative models, the proposed framework demonstrates notably enhanced accuracy in delineating both megakaryocyte nucleus and cytoplasm boundaries. Theera-Umpon’s (23) study showcased an automated segmentation technique tailored for bone marrow leukocyte micrographs, yielding commendable outcomes in both whole cell and nucleus segmentation. Moreover, extreme learning machine (ELM) classification methods, coupled with the generation of white blood cell (WBC) binary masks derived from precise segmentation via the CNN U-Net, are frequently employed for bone marrow cell segmentation (15, 24). Additionally, a robust segmentation technique has been developed to eliminate red blood cells and background noise from blood microscopy images, subsequently segmenting white blood cells within the remaining regions. Experimental findings demonstrate the effectiveness of this proposed automatic robust method in accurately segmenting leukocytes across various types of blood micrographs, surpassing other existing methods in terms of both robustness and accuracy (25).

## Automation of bone marrow cell morphology identification

**FIGURE 1:**
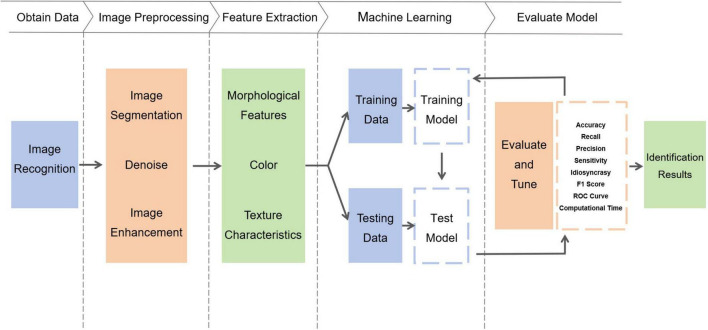
Schematic flowchart of machine learning automated identification system.

Accurate identification and characterization of bone marrow cell morphology is critical to the diagnosis of hematologic disorders. Nonetheless, the subjective and time-intensive manual identification process conducted by pathologists or hematologists poses challenges to prompt diagnosis and patient treatment. To address this issue, numerous studies have developed automated bone marrow cell recognition systems based on machine learning, (Figure 1) offering commendable stability, high accuracy, and robustness (26). Recent evaluations in medical image recognition underscore the remarkable capabilities of deep learning in automatically recognizing various bone marrow cell types and analyzing extensive image datasets, thus furnishing reliable classification outcomes (6, 27).

Eckardt et al.’s (27) study introduced an algorithm employing deep learning techniques to automatically distinguish hematopoietic cell lines and identify dysplastic cells in bone marrow aspiration smears. This algorithm serves as a diagnostic aid, aiming to reduce diagnostic time and standardize visual interpretation (28). Furthermore, a multi-stage deep learning platform has been documented for automatically analyzing bone marrow smear images. This platform has demonstrated the capability to discern the presence of acute promyelocytic leukemia solely through visual image data, showcasing the potential of deep learning in detecting diverse morphologies associated with cytogenetic abnormalities in leukemia.

Rosenberg et al. (29) demonstrated a novel label-free approach that integrates image recognition and Raman spectroscopy to swiftly identify nucleated red blood cells in blood samples. Concurrently, their research revealed that the visual object detection deep learning algorithm YOLOv3 exhibits superior real-time detection capabilities on economical computer setups for recognizing cell morphology, albeit with a lower precision (17). Imaging flow cytometry, as a robust and objective tool, enables the simultaneous analysis of phenotypic parameters alongside image-based morphological features like cell size and nuclearity, thereby potentially mitigating inter-observer discrepancies. Moreover, leveraging machine learning algorithms, imaging flow cytometry proves adept at precisely identifying binucleated erythrocytes, thus illustrating the feasibility of automated imaging flow cytometry-based approaches in investigating and quantifying morphological alterations in bone marrow cells.

The results of several studies have shown that Morphogo, a morphological examination system based on deep learning CNNs, is capable of high-precision automated identification of bone marrow cells using digital imaging and cellular histology analysis with an overall accuracy of 99%. This algorithm incorporates geometric cytological attributes, color, and texture features derived from various lymphocyte images as a training dataset, thereby expanding the scope and enhancing the accuracy of the automated digital microscopy system for lymphocyte identification in bone marrow (3, 26). Findings from several investigations have emphasized the clinical utility of Morphogo and its significance in aiding pathologists or hematologists in diagnosing blood disorders (26). The adoption of this system is poised to drive forward the automation and standardization of bone marrow cytology practices (30).

An automated morphological analysis system has been documented as effective in distinguishing various blood cells in blood images and assessing the morphological characteristics of these cells. Another study proposed the utilization of a novel high-performance end-to-end convolutional deep architecture, WBCsNet, which outpaces pre-trained counterparts in speed, as a pre-training network for visual feature extraction. This approach yielded a heightened overall accuracy in leukocyte recognition compared to alternative transfer learning methods and conventional recognition systems (31). In addition, a robust segmentation technique developed in a study was applied to identify malignant leukocytes in blood samples by extracting features including shape, color, and LBP-based texture features according to the visual criteria of hematologists (25).

## Automation of bone marrow cell morphology classification

The classification of Bone Marrow Cell Morphology stands as a pivotal cornerstone in hematologic diagnosis (32). Nonetheless, several challenges persist in this classification process. These challenges include (1) the intricate nature of myeloid cell growth and differentiation, characterized by multiple stages of development. (2) The presence of intra-class similarities among bone marrow cells at different growth stages within the same broad classification (e.g., granulocytes), as well as inter-class similarities among bone marrow cells at the same growth stage but belonging to different broad classifications (e.g., erythroid and lymphoid bone marrow cells) (6). Presently, cell morphology classification predominantly relies on experienced operators, a method fraught with limitations such as variability in speed and accuracy influenced by factors like operator proficiency and fatigue levels (16). Thus, there arises a pressing need for automated classification systems that are both cost-effective and efficient diagnostic aids, capable of replacing manual methods for the classification of bone marrow cells.

In recent years, deep learning frameworks have proven to be both successful and efficient in various tasks involving the processing of digital histology images. Among these tasks, cell categorization has emerged as a pivotal component in computer-aided diagnostic systems (13). Leveraging a training dataset comprising bone marrow puncture cell components, a recent study devised a machine learning algorithm employing a two-stage detection and classification approach to accurately categorize bone marrow puncture cells. During sixfold cross-validation, the algorithm demonstrated promising initial outcomes, exhibiting high overall accuracy when applied to non-tumor samples for classification. Consequently, this system represents an initial stride toward devising a dependable and objective automated method for conducting differential cell counting, with potential implications for disease diagnosis and prognosis in clinical practice (11).

Accurate classification of bone marrow cells is difficult due to the inhomogeneity and lack of reproducibility of differential counts. To address this, an automated classification system based on deep learning has been developed. Semi-supervised learning, which learns while labeling, has been employed to mitigate the requirement for a large volume of precisely labeled datasets for training purposes. Nakamura et al. (33) showed that the combination of confirmed self-training + active learning with two semi-supervised learning methods can effectively increase the training data for the development of an automatic classification system for bone marrow cells. Traditionally, cell detection and classification involve the use of two separate deep learning networks in a sequential manner, leading to increased computational complexity during the training phase.

However, Song et al.’s (13) study proposed a novel approach utilizing a simultaneous deep self-coder network with a unified architecture for the concurrent detection and classification of cells in bone marrow histology images. A curve-supported Gaussian model and a neighborhood selection mechanism are utilized to enhance the accuracy of detecting and classifying irregularly shaped cells. Consequently, this network demonstrates superior competitiveness compared to traditional deep learning classification networks in both performance and runtime efficiency. Furthermore, in a separate study, Wang et al. (12) introduced an efficient and fully automated hierarchical deep learning framework for classification-based analysis. This framework is capable of swiftly analyzing bone marrow nucleated difference counts on entire images (BM NDC WSI) within seconds, while also integrating multi-type plaque detection. When tested using cross-validation methods, the learning framework exhibited outstanding performance, achieving significant recall rates and over 90% accuracy, surpassing existing benchmark methods reliant on smaller image datasets. Future research endeavors aim to address challenges related to overlapping plaques, as the current framework does not incorporate mismatch classification.

The study by Huang et al. (5) demonstrates that a CNN combined with a transfer learning-based leukemia cell morphology classification method using bone marrow cell microscopy images is both feasible and efficient for classifying small sample datasets. This approach helps to mitigate errors, misdiagnosis, and misclassification stemming from human factors. By employing a combination of simplified image preprocessing and transfer learning, the classification accuracy of the model can be improved, enabling the differentiation of bone marrow samples from individuals with different types of leukemia as well as healthy subjects. Therefore, image preprocessing holds promise for enhancing medical image classification tasks, particularly when external factors such as acquisition equipment, lighting conditions, and noise significantly impact the image quality. The results of Shafique and Tehsin (14) showed that the deployment of AlexNet, a pre-trained deep CNN that does not require micrographic image segmentation, as opposed to training from scratch, resulting in a sensitivity, specificity, and accuracy of more than 96% in the classification of subtypes of acute lymphoblastic leukemia. Furthermore, to mitigate overfitting, the study employed data augmentation techniques. Matek et al. (32) applied CNNs to the most extensive expert-annotated dataset of bone marrow cytology images available in the literature. Their study successfully trained a high-quality leukocyte cytomorphology classifier, offering a proof-of-concept for the challenging task of classifying individual bone marrow cells. The neural network developed in their research utilizes cutting-edge image classification algorithms to automatically evaluate bone marrow cell morphology, surpassing previous machine learning methods in terms of accuracy and generalizability.

In a separate investigation, Kutlu et al. (2) proposed a computer-aided automatic system based on regional convolutional neural networks (R-CNN) to simultaneously classify different cell types within the same image. The dataset utilized in this study serves as a valuable reference for future developments in AI-based bone marrow cell morphology methodologies. Another study introduced a bone marrow cell classification algorithm based on a hierarchical network (Hierarchical-CNN) model tailored to the characteristics of bone marrow cells. This algorithm achieves automatic classification of individual bone marrow cell images by employing a two-stage process for classification and recognition, significantly enhancing the accuracy of both classification and recognition tasks. The initial stage involves training a classification network (Parent DCNN) to classify and identify all categories of bone marrow cells. Subsequently, the second stage employs two sub-networks: the M-M DCNN, which learns intra-class differences, and the E-LDCNN network, which discerns both intra- and inter-class disparities. Utilizing a deep residual network, ResNet, as the foundational network structure enabled comprehensive feature parameter learning of bone marrow cells, resulting in commendable classification and recognition accuracy (6).

Furthermore, Devidas Pergad and Hamde (34) proposed a novel neural network (NN) classifier integrated with the fractional gravitational search (FGS) algorithm for weight updating in radial basis function mapping to classify leukocytes based on their nuclear features. Evaluation results indicate that the highest accuracy is attained solely through nuclear features for classification. Additionally, the FGS-RBNN classifier demonstrates superior classification performance compared to existing methods, achieving an accuracy rate as high as 95%. In a separate study, Ravikumar (24) demonstrated the effective and efficient segmentation and classification of leukocytes using Extreme Learning Machines and Fast Correlation Vector Machines.

In contexts involving high-risk systems like bone marrow cell identification, conventional classification machine learning models often exhibit significant limitations as they solely produce classification outcomes without the ability to abstain from making predictions when prediction reliability is low. To tackle this challenge, a CNN, ICP, and SoftMax (CNN-ICP-SoftMax) based method known as Classifying Myeloid Cells with Rejection Option (CMWRO) has been proposed specifically for myeloid cell classification. With a rejection rate (RR) of 0.3 for test samples, the CMWRO ensures the acceptance of samples with a satisfactory 0.9 precision, sensitivity, and accuracy. Samples automatically rejected by the system are subsequently processed by alternative means, such as manual identification by a hematologist. Notably, the method demonstrates effective filtering of cell types for which the classifier has not been trained, including abnormal cells and those with limited sample distribution, achieving a filtering efficiency of over 82%. The implementation of CMWRO has notably bolstered doctors’ confidence in model outputs. Physicians can now concentrate solely on samples rejected by CMWRO, integrating computer and manual identification results to arrive at a final diagnosis. This approach significantly streamlines the diagnostic process, rendering it more efficient, accurate, and dependable (35).

Ghane et al. (36) proposed a straightforward and efficient computer-aided diagnostic (CAD) method based on microscopic image processing. Their approach introduces a novel combination of both typical and innovative features aimed at classifying chronic myeloid leukemia (CML) cells and categorizing bone marrow cells through an effective decision tree classifier. The proposed classification method boasts an accuracy, specificity, consistency of results, and sensitivity exceeding 98%. Consequently, this CAD method exhibits robust classification capabilities and can serve as a straightforward, cost-effective, and dependable tool for diagnosing chronic granulocytic leukemia. In a separate investigation, Agaian et al. (37) developed a comprehensive automatic classification system for whole-image analysis. Their system relies on cellular energy and color features to automatically categorize peripheral blood smear images of acute lymphoblastic leukemia (ALL) characterized by multiple nuclei. The results obtained from the system performance evaluation, conducted through multiple cross-validation methods, demonstrate the efficiency and effectiveness of the proposed system in accurately classifying acute leukemia cells in blood smear images.

In efforts to streamline the intricate process of leukemia cell classification within existing point-of-care testing (POCT) devices, Lv et al. (38) devised a leukocyte classification detection system leveraging microfluidics and multimodal imaging. This innovative system boasts advantages such as compact size, user-friendly operation, rapid single-sample detection, high accuracy, and minimal maintenance requirements. Notably, the system achieves automatic leukocyte classification through the implementation of a BP neural network model.

In a separate investigation, Fanous et al. (16) introduced a methodology for the automatic detection and classification of leukocytes utilizing microscopic color images and neural classifiers. Furthermore, Piuri and Scotti (15) demonstrated the feasibility of leukocyte classification and analysis using phase imaging with Computational Specificity (PICS). Additionally, Fu et al. (39) developed the AI-based Morphogo system, aimed at the automated classification of bone marrow cells and identification of their potential clinical applications. This system achieves an accuracy exceeding 85.7% in classifying hematopoietic lineage cells.

Furthermore, cooperative games have been leveraged to categorize markers based on varying assigned weights, suggesting the potential applicability of game theory for direct classification in diverse contexts. To this end, Torkaman et al. (40) have introduced an automatic leukemia classification system grounded in game theory principles, yielding a classification accuracy of 98.44% (Table 1).

**TABLE 1:** A summary of examples of BMC classification automation.

References	Purpose	Number of images/cells	Highlight	Advantages
Chandradevan et al. (11)	BMC detection and classification	9,269 annotated cells	A two-stage detection and classification approach that enables design flexibility and improves classification accuracy.	Higher accuracy
Nakamura et al. (33)	BMC classification	68,238 single cell images	Self-training + active learning combined with two semi-supervised learning methods.	Higher accuracy and ability to efficiently increase training data
Song et al. (13)	BMC detection and classification		A synchronized deep autoencoder network for simultaneous detection and classification of cells in bone marrow histology images and using a neighborhood selection mechanism to improve the classification accuracy.	Higher accuracy, better performance, and shorter training time
Wang et al. (12)	BMC detection and classification		An efficient and fully automated hierarchical deep learning framework.	Higher recall and accuracy, and shorter computational time
Huang et al. (5)	BMC classification and diagnosis	1,322 BMC images	The perfect reflection algorithm and a self-adaptive filter algorithm were first used for preprocessing of bone marrow cell images collected from experiments. Three CNN frameworks (Inception-V3, ResNet50, and DenseNet121) are combined to construct classification models for the raw dataset and preprocessed datasets. Transfer learning technique is used to improve the prediction accuracy of the model.	Higher accuracy and speed
Shafique and Tehsin (14)	BMC detection and classification	500 leukemia + 260 normal	Fine-tune the dataset using the pretrained AlexNet. Replace the last layer of the pretrained network with new layers that classify the input images into four classes.	Higher sensitivity, specificity, and accuracy
Kutlu et al. (2)	BMC detection and classification	6,250 WBC images	Different cell types within the same image have been classified simultaneously with a detector by regional convolutional neural networks.	Higher accuracy
Matek et al. (32)	BMC identification and classification	171,374 single-cell images from 945 patients	Applying convolutional neural networks to a large data set of microscopic cytological images.	Higher precision and recall
Shi (6)	BMC identification and classification	15,309 single BMC images from 3,122 patients	Bone marrow cell classification algorithm based on Hierarchical-CNN model for automatic classification of individual bone marrow cell images.	Higher accuracy
Devidas Pergad and Hamde (34)	WBC classification		An NN classifier using the integrated fractional gravitation search algorithm for updating the weight in the radial basis function mapping for the classification of the WBC based on the cell nucleus feature.	Higher accuracy, sensitivity, and specificity
Ravikumar (24)	Leukocyte segmentation and classification		To achieve the maximum accuracy of the RVM classifier, we design a search for the best value of the parameters that tune its discriminant function, and upstream by looking for the best subset of features that feed the classifier. Designing the search for the parameters of the RVM classifier to find the maximum accuracy.	Higher accuracy and shorter calculation times
Guo et al. (35)	BMC classification		Bone marrow cell classification with rejection option based on convolutional neural networks, ICP and SoftMax.	Higher precision, sensitivity, and accuracy
Ghane et al. (36)	Chronic myeloid leukemia cell classification	1,730 WBC	Introduction of typical and new features in computer-aided diagnostic methods based on microscopic image processing.	Higher accuracy, specificity, and sensitivity and consistency of results
Agaian et al. (37)	Classification of peripheral blood smear images in acute lymphoblastic leukemia		Whole image automated classification system based on cell energy, color features.	More efficient
Lv et al. (38)	Leukocyte classification	22,092 WBC data	Leukocyte classification detection system based on BP neural network model and microfluidics and multimodal imaging.	Small size, simple operation, fast single-sample detection, high accuracy, and maintenance-free
Fu et al. (39)	BMC classification	65,986 BMCs from 230 BM smears	Morphogo on digital images analyzed by AI.	Higher accuracy

## Automation of bone marrow cell morphology enumeration

Currently, the differential counts and sizes of various leukocyte types play a crucial role in assessing a range of significant hematologic pathologies (4). However, cell counting encounters several challenges, including (1) variations among cells at each stage, (2) minimal interclass variation, (3) image discrepancies due to diverse collection and staining processes, (4) the need for sorting numerous cell types in bone marrow smear analysis, and (5) difficulties in individual cell counting arising from the high density of contact cells (41). Given these challenges, the manual detection of bone marrow cells, the current standard reference method for determining differential counts, presents drawbacks such as subjectivity, crudeness, and cumbersome procedures (42). Moreover, it often leads to inconsistencies and discrepancies among hematologists’ results (41). Consequently, there is a growing interest in research and development focusing on machine learning-based automation systems for cell counting.

In recent years, numerous experiments have underscored the potential of deep learning methods in revolutionizing bone marrow cell counting, owing to their remarkable attributes of high accuracy and speed (42). For instance, van Eekelen et al. (43) demonstrated the application of digital image analysis grounded in deep learning for quantifying bone marrow cell numbers more objectively. Likewise, a reticulocyte counting model trained to utilize the Faster R-CNN deep neural network showcased precise and objective cell counting capabilities (42).

Baranova et al. (44) reported in their study that QuPath, a validated open-source digital pathology tool, can effectively and automatically quantify and enumerate the number and percentage of CD138-positive bone marrow plasma cells. Particularly noteworthy is its accuracy in cases where the percentage of these cells was below 30%, showing a strong correlation with pathologist-derived counts through image analysis.

Furthermore, Choi et al. (41) conducted a study wherein they introduced an automated leukocyte differential counting system tailored for bone marrow smear images, employing a two-stage CNN. Notably, the system exhibited not only high classification performance but also achieved accuracy, recall, and F-1 scores surpassing 97%. These findings underscore the potential of two-stage CNNs as an effective automatic leukocyte classification and counting system.

## Automated bone marrow cell morphology diagnostic

Despite advancements in new molecular markers and prognostic tools, manual bone marrow histomorphometric analysis persists as a foundational and essential gold standard, playing a mandatory role in aiding the identification, diagnosis, and assessment of outcomes for hematologic disorders (19, 45). Nonetheless, manual cytomorphometric analysis suffers from several drawbacks, including being time-consuming, repetitive, subjective, reliant on hematologist experience, and susceptible to inter- and intra-specialist variability (45). Hence, there exists a pressing demand for an automated diagnostic system capable of swiftly, accurately, and objectively analyzing bone marrow cytomorphology (5). In recent years, the utilization of deep learning models has surged in the diagnosis of medical image problems (45), particularly in aiding hematologists in expediting diagnosis and disease monitoring through the interpretation of bone marrow smears (19). Leveraging vast annotated morphological data, CNN image recognition techniques empower the training of AI algorithms for diagnosis, enabling real-time, rapid, and objective morphological analysis of specimens (45). Moreover, computer-aided systems can aid specialists in disease diagnosis, thereby reducing the likelihood of inappropriate treatments (46).

The study introduces an efficient, fast, and scalable preliminary framework for diagnosing hereditary hemolytic anemia samples, utilizing automated rheology and machine learning. Employing state-of-the-art algorithms, the framework adeptly distinguishes between healthy populations and patients, demonstrating high accuracy in identifying various diseases. Moreover, it efficiently conducts initial identification or differentiation of complex diseases even with limited sample sizes (8).

Unlike conventional machine learning methods, CNNs are favored for medical image analysis due to their automatic feature learning and representation capabilities. Nevertheless, the exceptional performance of CNNs hinges on substantial time, cost, and computational resources, alongside an abundance of high-quality training data samples. To extend the applicability of deep learning in medical imaging, it has become common practice to fully exploit existing small-sample medical image datasets through a combination of data augmentation and transfer learning. This approach is not only rapid and efficient but also yields objective and reliable results, capable of discerning subtle morphological changes imperceptible to the naked eye, thereby significantly enhancing diagnostic accuracy. This methodology is exemplified in the study conducted by Huang et al. (5). A CNN augmented with transfer learning was employed to devise an assisted diagnostic approach aimed at replacing the manual interpretation of bone marrow cell morphology through the utilization of bone marrow cell microscopy images. Furthermore, this method offers the potential to standardize the diagnosis of bone marrow smears by mitigating the subjective factors inherent in manual interpretation. Wu et al.’s (19) study introduced a deep learning model (BMSNet) constructed on the YOLO v3 architecture, designed to assist hematologists in interpreting bone marrow smears by facilitating the rapid detection of hematopoietic cells, thus expediting diagnosis and disease monitoring. However, the current BMSNet model struggles to accurately identify aberrant features of hematopoietic cells, posing challenges in detecting minimal residual disease (MRD) levels. Hence, the outcomes should undergo thorough scrutiny and detailed morphological interpretations by seasoned hematologists to ensure result consistency before the AI interpretation translates the findings into clinical data. The trained AI model BMSNet not only exhibits the capability to accurately and swiftly detect the percentage of progenitor cells, estimating the severity of leukemia and treatment efficacy but also aids hospitals lacking hematologists in interpreting bone marrow smears. In a study by Xiao et al. (45), the AI cell morphology analysis system consistently demonstrated a positive correlation with the proportion of primitive cells analyzed by hematologists (*r* > 0.8, *P* < 0.001), irrespective of treatment. Utilizing expert analysis results as the benchmark, the AI cell morphology analysis system exhibited high accuracy, sensitivity, and satisfactory specificity in identifying primitive cells during the morphological diagnosis of acute myeloid leukemia and assessing treatment efficacy. Future research endeavors should further explore the clinical utility of this system.

## Discussion

Efficient and robust cell detection stands as a pivotal step in numerous computer-aided diagnostic systems designed for processing digital histology images (13). However, this task poses persistent challenges due to factors such as cells being closely juxtaposed, heterogeneous background noise, and considerable variability in cell size and shape (10) (Table 2). CNNs applied to bone marrow cell morphology represent deep learning models adept at automating the detection of bone marrow cells in bone marrow aspirate smears. Notable examples include BMSNet (19), Faster R-CNN (42), and AlexNet (14). Leveraging CNNs in bone marrow cell morphology analysis can significantly expedite hematologists’ interpretation of bone marrow smears, facilitating swifter diagnosis and disease monitoring. Moreover, such technologies hold promise in aiding disease prognosis, thereby significant impact on clinical practice. Furthermore, the utilization of innovative techniques like fast correlation vector machines (24), the Morphogo system (30), and color spatial light interference microscopy coupled with deep learning tools (15) is anticipated to propel the automation and standardization of bone marrow smear examination. Deep learning methodologies boast remarkable attributes such as precision, recall, heightened sensitivity and specificity, and efficiency. Nevertheless, detailed morphological interpretation still necessitates the expertise of trained hematologists. Consequently, the fusion of digital pathology imaging with machine learning algorithms represents a promising frontier in emerging technology (44).

**TABLE 2:** Comparison table of advantages and disadvantages of machine learning methods for BMC applications.

Algorithm name	Advantage	Disadvantage
Deep learning CNNs	1. Effective capture of localized features 2. Abstraction of high-level semantic features 3. Strong automatic feature learning and presentation skills 4. High precision, sensitivity, specificity, and accuracy	1. Requires significant time, cost, and computational resources 2. Requires large-scale, high-quality training data
Decision tree RF	1. Strong generalization ability 2. Insensitivity to missing data 3. Handling high-dimensional data 4. Feature selection and importance assessment 5. Highly parallelized training	1. High computational and memory requirements 2. Sensitive to noise and outliers 3. Sensitive to different value attributes
Support vector machine (SVM)	1. No need to rely on the entire dataset 2. Handling nonlinear features 3. Solving high-dimensional problems 4. The problem of no local minima 5. High efficiency in segmentation and categorization	1. Inefficient when observing a large sample 2. No generalized solution for nonlinear problems 3. The kernel function has little explanatory power 4. Supports only two classifications 5. Sensitivity to missing data

This table summarizes the advantages and disadvantages of deep learning CNN, decision tree RF, and support vector machine (SVM) for BMC morphology applications. Each algorithm has its unique advantages and application scenarios, and choosing the right algorithm needs to be decided based on specific needs and data characteristics.

Accurate segmentation of bone marrow cells presents a considerable challenge owing to the intricate composition of bone marrow smears. Nonetheless, this segmentation is pivotal for disease identification and aids experts in diagnosing blood disorders. Therefore, as an important tool for disease diagnosis in the medical field, the development and application of automated segmentation systems have become a hot research topic in recent years. In a study by Su et al. (18), segmentation of nuclei employed stepwise averaging and the Otsu method. For cytoplasmic segmentation, techniques such as color weakening transformation, an enhanced region-growing method, and the K-Means algorithm were utilized. The separation of connected cells, particularly myeloid cells, was achieved through the labeling control watershed algorithm. Notably, several studies (34, 38) have highlighted the superior performance of new neural network classifiers in terms of accuracy, sensitivity, recall, and specificity compared to existing classifier methods.

In recent years, assessments of deep learning in medical image recognition have underscored its remarkable capacity to analyze vast amounts of image data and yield dependable classification outcomes based on objective quantitative criteria. Notably, Morphogo, a morphological examination system founded on CNNs, has demonstrated proficiency in utilizing digital imaging analysis to discern over 25 distinct types of bone marrow cells with specificity and an overall accuracy exceeding 99% (26). Intriguingly, algorithms integrating geometric cytological features, color, and texture features of bone marrow cell images have been shown to broaden the scope and enhance the accuracy of the system’s recognition capabilities (3). These findings serve to corroborate the efficacy of the Morphogo system in clinical settings and underscore its significance in aiding pathologists in diagnosing blood disorders. It is worth noting that AI-based cell morphology analysis techniques exhibit superior accuracy, sensitivity, and specificity in bone marrow cell morphology recognition for diagnosis and treatment compared to traditional methods (45).

The classification of bone marrow cell morphology stands as a pivotal aspect of hematological diagnosis, furnishing insights into the distribution of bone marrow cells across various stages. Such insights are instrumental in diagnosing a spectrum of diseases and guiding post-chemotherapy follow-up care. Hence, the development of a deep learning system for bone marrow cells is of paramount importance. Presently, CNNs are increasingly deployed in the classification and diagnosis of medical image issues (2, 14, 41). In addition, automatic myeloid cell classification systems include myeloid cell classifiers with rejection options (35), computer-aided diagnostic methods based on microscopic image processing (36), AI-based automated analysis systems (Morphogo) (39), novel NN classifiers for classifying myeloid cells based on their nuclei features (34), microfluidics-based and multimodal imaging for classification and detection system (38), etc. The classification efficacy of the aforementioned systems, as gauged by metrics like accuracy, recall, sensitivity, specificity, and F-1 score, surpasses that of existing classifiers. However, the training and classification of limited small datasets pose a significant challenge for CNN-based deep learning systems. To surmount this hurdle, transfer learning is recommended for extracting image features to facilitate subsequent classification (5).

Bone marrow differential count serves as a pivotal assessment parameter and diagnostic test for a spectrum of blood diseases, holding significant clinical value in disease classification, monitoring therapeutic outcomes, and predicting prognosis. Within the realm of bone marrow examination, the analysis of bone marrow nuclear differentials stands out as paramount, offering fundamental and crucial insights. However, the accurate classification of bone marrow cells proves challenging due to the heterogeneity and lack of repeatability inherent in the differential count process. Presently, deep learning methodologies, including the Faster R-CNN deep neural network (42), the open-source digital pathology tool QuPath (4), the convolutional deep architecture “WBCsNet” (22), and the Morphogo system (46), exhibit promising potential as rapid computer-aided solutions for manual bone marrow cell counting. These systems have already undergone validation as reliable tools for automated bone marrow cell counting analysis, showing promise for clinical applications. However, the necessity for future large-scale multicenter validation studies is evident, as these endeavors will furnish additional insights to further affirm the clinical utility of these systems. It is notable that compared to other areas covered in this review, there has been relatively less research focused on automating bone marrow cell counting. Moving forward, there is a pressing need to ramp up efforts in developing and validating automation algorithms and models for cell counting in the bone marrow.

Bone marrow cytomorphometric analysis stands as a fundamental and pivotal test aiding in the diagnosis, differential diagnosis, and efficacy assessment of hematologic diseases. The integration of deep learning models offers hematologists a tool for expediting the interpretation of bone marrow smears, facilitating swift diagnosis and disease monitoring (19), while concurrently mitigating the risk of inappropriate treatment prescriptions (5). Through the amalgamation of digital pathology and machine learning, novel diagnostic features and algorithms can be extracted from digital bone marrow cell images, culminating in the development of a robust and expeditious screening and diagnostic tool (3). AI cell morphology analysis system has a high accuracy and sensitivity and good specificity for cell identification in leukemia morphology diagnosis and efficacy assessment (45). However, there are still few research results that can be referred to in the field of bone marrow cell morphology testing and diagnosis, and more research is needed in the future to explore the value of the clinical application of AI in bone marrow cell morphology.

In recent years, along with the enhancement of deep learning methods, machine learning algorithms, and big data analysis, AI technology based on machine learning has ushered in a development boom (47) and provides powerful assistance for clinical diagnosis. With the emerging trend of integrating computer-aided diagnostic tools into both laboratory and clinical medicine, there has been a surge of interest in the development, training, and validation of novel machine-learning-based models and algorithms. These innovations aim to automate and standardize myeloid cytology and disease diagnosis, representing a compelling new frontier in research (48).

In the foreseeable future, automated systems driven by machine learning are anticipated to significantly enhance the experiences of both clinicians and patients in bone marrow cell morphology testing and diagnosis (49). These systems hold the potential to gradually supplant manual microscopy examinations, leading to breakthroughs in bone marrow cell morphology testing and diagnosis. Moreover, the integration of machine-learning automated systems with clinical practice, facilitating the provision of diagnostic reports for peripheral blood cytology, replete with rich graphical interpretations, precise results, and lucid diagnoses, has emerged as a prominent focus of recent research endeavors (48). In the analysis of bone marrow cell morphology results, a hybrid approach combining automated analysis and manual review mode will be essential for enhancing the accuracy of machine learning-assisted assistance, as well as improving the efficiency of laboratory personnel and fostering trust in the results (35).

Artificial intelligence holds significant promise in enhancing the workflow efficiency of bone marrow cell morphology testing. The analysis of genome-associated bone marrow cell morphology changes through the training of deep learning models enables rapid and accurate diagnosis of acute promyelocytic leukemia, a critical aspect of patient clinical management. Constructing a leukemia bone marrow morphology evaluation model utilizing the cloud platform’s leukemia big data analysis algorithm facilitates the establishment of a patient’s database. Simultaneously, it enables the development of an automatic leukemia risk assessment model and the implementation of remote consultations, contributing to early screening and treatment of blood diseases at the grassroots level. This approach not only reduces personal and national health insurance costs but also aligns with the policy of hierarchical diagnosis and treatment.

In the future, further optimization of algorithms and model enhancement is essential. This includes refining the algorithm, improving the model, and expanding training and validation processes to incorporate more samples and multi-center datasets. Additionally, designing a few-sample learning method to effectively learn from rare samples is imperative. Experts emphasize the need to establish AI-assisted diagnostic standards, specifications, and expert consensus on bone marrow cell morphology. They advocate for the creation of an authoritative, scientific, standardized, and diversified morphology database. Furthermore, they stress the importance of establishing an AI-assisted morphology recognition, cell classification, and quality evaluation system, utilizing either a large field of view or a full-picture image. Additionally, the establishment of a nationwide clinical case discussion platform and remote diagnosis platform for cytomorphology testing and diagnosis is recommended, thereby promoting the realization of bone marrow cytomorphology remote consultation.

Experts emphasize the importance of addressing four key issues concerning standardization and enhancement within the field: (1) Development of standardized AI-assisted diagnostic criteria, norms, and expert consensus for blood cell morphology. (2) Creation of authoritative, scientific, standardized, and diverse morphological databases. (3) Establishment of an AI-assisted quality evaluation system for morphology recognition, cell classification, and cytomorphology testing and diagnosis based on large field of view or whole picture images. (4) Establishment of a nationwide network platform for clinical case discussions and remote diagnosis in cytomorphological testing, aimed at elevating the standard of hemocyte morphology diagnosis (48).

## Conclusion

In summary, traditional manual methods of analyzing bone marrow cell morphology have demonstrated limitations across various dimensions. To address these shortcomings, automating the examination and analysis processes, which still rely heavily on manual intervention, emerges as the most viable solution. Urgently needed are automated methods offering objectivity, efficiency, standardization, and reproducibility. Automation of these steps will have a significant and far-reaching positive impact on diagnosis and patient outcomes for hematologists. Therefore, it is crucial to further summarize the current status and progress of research on the automation of machine learning in the examination and analysis of bone marrow cell morphology.

## Author contributions

YL: Writing – original draft, Writing – review & editing. QC: Writing – review & editing. TC: Conceptualization, Methodology, Writing – review & editing.
